# Identifying interventions with Gypsies, Roma and Travellers to promote immunisation uptake: methodological approach and findings

**DOI:** 10.1186/s12889-020-09614-4

**Published:** 2020-10-20

**Authors:** Lisa Dyson, Helen Bedford, Louise Condon, Carol Emslie, Lana Ireland, Julie Mytton, Karen Overend, Sarah Redsell, Zoe Richardson, Cath Jackson

**Affiliations:** 1 Department of Health Sciences, University of York, Heslington, York, YO10 5DD UK; 2 UCL Great Ormond Street Institute of Child Health, 30 Guilford Street, London, WC1N 1EH UK; 3 College of Human and Health Sciences, Swansea University, Singleton Park, Swansea, SA2 8PP UK; 4 School of Health & Life Sciences, Glasgow Caledonian University, Cowcaddens Road, Glasgow, G4 0BA UK; 5 University of the West of England, Centre for Child and Adolescent Health, Oakfield House, Oakfield Grove, Bristol, BS8 2BN UK; 6 Faculty of Health, Social Care and Education, Anglia Ruskin University East Road Campus, Cambridge, CB1 1PT UK

**Keywords:** Co-production, Gypsy, Immunisation, intervention development, Roma, Traveller

## Abstract

**Background:**

In the UK, Gypsy, Roma and Traveller (GRT) communities are generally considered to be at risk of low or variable immunisation uptake. Many strategies to increase uptake for the general population are relevant for GRT communities, however additional approaches may also be required, and importantly one cannot assume that “one size fits all”. Robust methods are needed to identify content and methods of delivery that are likely to be acceptable, feasible, effective and cost effective. In this paper, we describe the approach taken to identify potential interventions to increase uptake of immunisations in six GRT communities in four UK cities; and present the list of prioritised interventions that emerged.

**Methods:**

This work was conducted in three stages: (1) a modified intervention mapping process to identify ideas for potential interventions; (2) a two-step prioritisation activity at workshops with 51 GRTs and 25 Service Providers to agree a prioritised list of potentially feasible and acceptable interventions for each community; (3) cross-community synthesis to produce a final list of interventions. The theoretical framework underpinning the study was the Social Ecological Model.

**Results:**

Five priority interventions were agreed across communities and Service Providers to improve the uptake of immunisation amongst GRTs who are housed or settled on an authorised site. These interventions are all at the Institutional (e.g. cultural competence training) and Policy (e.g. protected funding) levels of the Social Ecological Model.

**Conclusions:**

The “upstream” nature of the five interventions reinforces the key role of GP practices, frontline workers and wider NHS systems on improving immunisation uptake. All five interventions have potentially broader applicability than GRTs. We believe that their impact would be enhanced if delivered as a combined package. The robust intervention development and co-production methods described could usefully be applied to other communities where poor uptake of immunisation is a concern.

**Study registration:**

Current Controlled Trials ISRCTN20019630, Date of registration 01-08-2013, Prospectively registered.

## Background

In the UK, Gypsy, Roma and Traveller (GRT) communities are generally considered to be at risk of low or variable immunisation uptake. This is based on data demonstrating low uptake of preventive health services [1–4], the findings of a small number of local studies assessing immunisation take up using self-report or National Health Service (NHS) records (e.g. [5, 6]), and publicised outbreaks of measles and whooping cough [6, 7]. Disease outbreaks amongst clusters of people pose a threat to wider community health even in countries where national and regional coverage is high [8].

The barriers to uptake by GRT communities reflect two groups of influencing factors for immunisation uptake in the general population and high-risk groups [9–11], namely access to services and beliefs about the necessity of vaccines or concerns about their safety. Although some differences exist between communities, our research [12–14] and other studies with diverse GRT communities [1–4, 15, 16] report that these groups encounter challenges accessing health services, including immunisation. These are often related to socio-economic and socio-cultural barriers for example, poverty, low literacy, discrimination leading to a lack of trust in health professionals, irregular school attendance, poor/changing housing conditions and language. The absence of a permanent postal address to register with a General Practitioner (GP) and difficulty securing prompt immunisation appointments are additional, practical, access barriers [12–14]. Studies exploring GRT views on immunisation report mixed acceptance [12–18], resistance to immunisations is associated with concerns about the potential side effects and/or a lack of belief in their value.

These multiple, complex and often interlinked factors mean that a broad range of approaches at individual, provider, health system, and national levels are needed to ensure optimum immunisation uptake. Many strategies that are developed and evaluated for the general population [19–22] are relevant for GRT communities, however additional or different approaches may be required. Importantly, one cannot assume that “one size fits all” because the cultural beliefs and established traditions of different GRT communities can vary [1–4, 12–14]. Robust intervention development is required to identify content and methods of delivery that are likely to be acceptable, feasible, effective and cost effective. Different approaches to intervention development exist and include guidance from the United Kingdom (UK) Medical Research Council (MRC) [23, 24], intervention mapping [25], behaviour change techniques [26] and guidelines specifically for tailoring immunisation programmes [27, 28]. In the UK and North America, there has been growing interest in applying co-production methods to public services such as health and social care [29]. Indeed, working with the target community to ensure that interventions and services are grounded in its views and experiences (process of co-production) is a hallmark of good quality care [30, 31]. Existing interventions with GRT communities focus on immunisation (e.g. pop-up immunisation clinics) or target preventive health more broadly (e.g. hand-held patient records) [1, 32–34]. However, they are rarely developed through exploring the multiple facets to GRT’s current behaviour in order to identify areas for potential intervention.

Our UNITING (UNderstanding uptake of Immunisations in TravellIng aNd Gypsy communities) study drew on the aforementioned guidance to systematically use evidence, theory, population segmentation and methods of co-production. It had two aims: (1) Investigate the barriers and facilitators to acceptability and uptake of immunisations amongst six GRT communities across four UK cities; (2) Identify potential interventions to increase uptake of immunisations in these GRT communities. The six communities were: Eastern European Roma and English Gypsy/Irish Traveller (Bristol), English Gypsy (York), Eastern European Roma and Scottish Showpeople (Glasgow), and Irish Traveller (London). The theoretical framework underpinning the study was the Social Ecological Model (SEM) [35] which recognises that the determinants of individuals’ behaviour are complex, multifaceted and operate at different levels (intrapersonal, interpersonal, institutional, community, policy). UNITING was a three-phase study. The methods and findings for the first two phases, interviews with GRTs and Service Providers, are presented elsewhere [12–14, 36, 37]. In this paper, we discuss the third phase of UNITING, the approach we took to identify potential interventions to increase uptake of immunisations in these GRT communities; and present the list of prioritised interventions that emerged.

## Methods

**Fig. 1:**
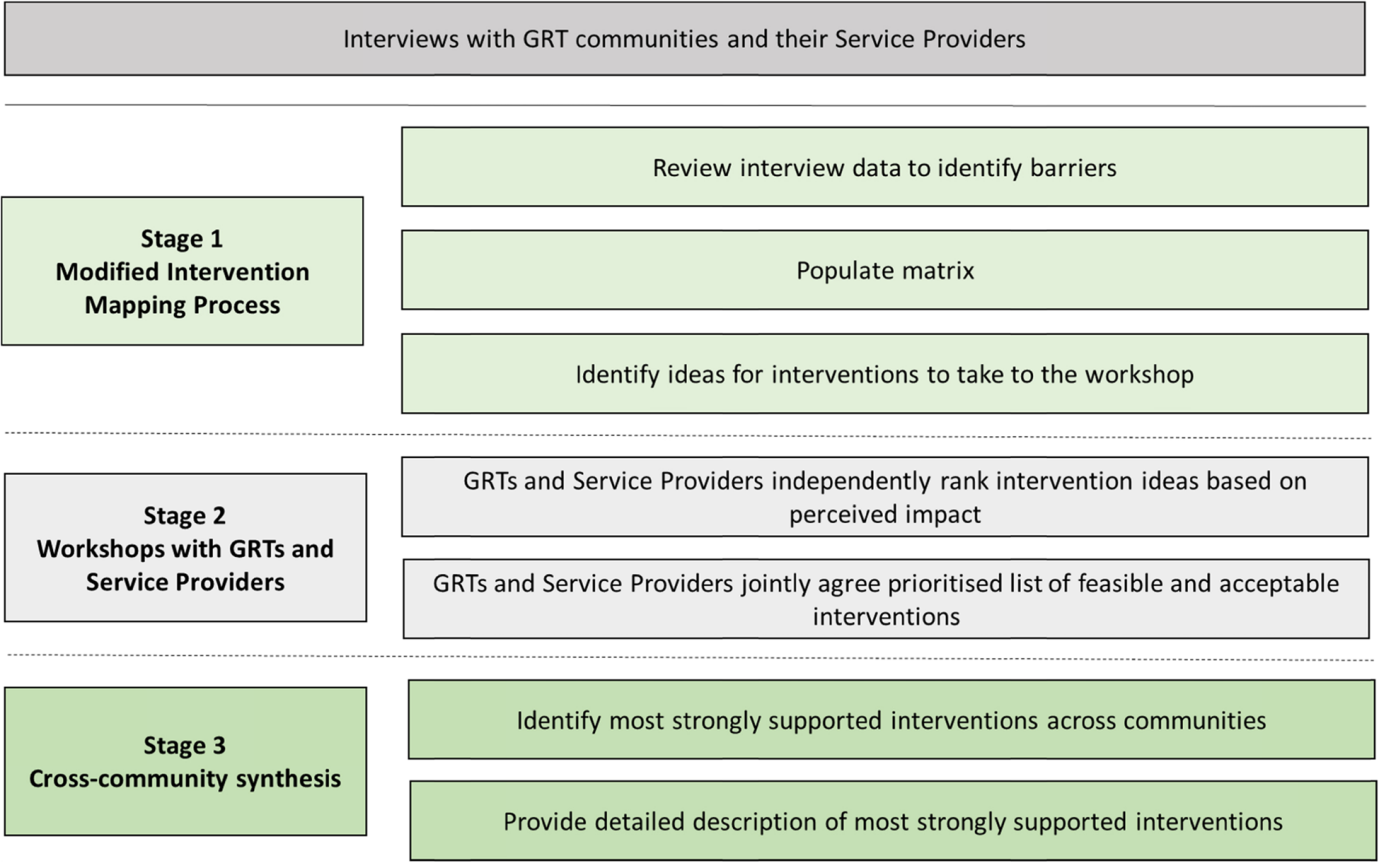
Stages of intervention development and assessment

This work was conducted in three stages (see Fig. 1). Each stage informed the next.

1. A modified intervention mapping process [25] using interviews previously conducted with GRTs and Service Providers [12–14] to identify ideas for potential interventions
2. Workshops with GRTs and Service Providers to agree a prioritised list of potentially feasible and acceptable interventions for each community
3. Cross-community synthesis to produce a final list of prioritised interventions

### Stage 1 – Modified intervention mapping process

A modified intervention mapping approach [25] was used to identify key ideas for interventions to be taken to workshops in Stage 2. This method uses theory and evidence to map “the path from recognition of a need or problem to the identification of a solution” [25]. The data from our interviews with GRTs and Service Providers [12–14] were used as evidence to identify the “need or problem” that could potentially be changed through intervention. Our focus was GRTs who were housed or settled on an authorised site because these were our interview participants. We did not recruit any GRTs living on the roadside or on unauthorised encampments to be interviewed. We sought to identify acceptable ideas for interventions at all five levels of the SEM [35], creating matrices which linked the barriers to immunisation, objectives to address each barrier, intervention target, SEM level and intervention ideas (see Table 1 for an example).

**Table 1:** Example from an intervention mapping matrix (Bristol English Gypsy/Irish Traveller community)

**Barrier**	**Concerns about specific vaccines**: *Whooping cough* - fear of brain damage in child, view that pregnancy should be natural *HPV* – belief that girls don’t need it as don’t have sex until married, so vaccine is seen as inappropriate and offensive	**Low levels of literacy:** meaning that information about immunisations is difficult to understand
**Objective to address barrier**	Develop good understanding of specific vaccines amongst GRTs whilst mindful of culturally-based concerns	Develop good understanding of immunisations amongst GRTs
**Target:**	Community Primary Care Schools	Community Primary Care
**SEM level**	Intrapersonal Interpersonal Institutional Community	Intrapersonal Institutional
**Ideas for Intervention**	Verbal explanation from a health professional^a^ Community Champions^a^ Social media with accurate messages^a^ Work with school nurses to change the way the HPV jab is presented to adolescent girls i.e. about cancer and when it is important to have it^b^ Cultural competence training; Work with targeted groups e.g. pregnant women, fathers, adolescent girls^c^ Support adolescent girls to speak with their elders^c^ Involve the community in developing culturally relevant information^c^	Explain things clearly and where information is written keep it simple using pictures^a,b^ Involve the community in developing accessible information^c^ Improve access/retention at school^c^

*Note.* ^a^Idea from GRTs, ^b^Idea from Service Providers, ^c^Idea from Research Team and Advisory Group

To generate as many potential interventions as possible, ideas were drawn from interviews with GRTs in the target community and their Service Providers, from interviews with GRTs from the other communities and their Service Providers, and from the knowledge and experience of the research team and advisory group. Two researchers independently mapped intervention ideas to the barriers, objectives, targets and SEM level and then jointly agreed the intervention ideas to take to the workshops. These fell into three categories: promoting awareness and understanding of immunisation, developing trust and respect, and improving immunisation services. The matrices produced for the first two communities (Bristol Eastern European Roma and Bristol English Gypsy/Irish Travellers) were used as a framework from which to build the matrices for the other communities, incorporating additional or adapted interventions based on the interviews. See Table 2 for the intervention ideas that were taken to the York workshop.

**Table 2:** Ideas for interventions taken to the York workshop with independent and jointly agreed rankings

Ideas for Intervention	English Gypsies’ rankings	Service Providers’ rankings^a^	Jointly agreed rankings
***Promoting awareness and understanding of immunisation***			
Good information from non-NHS sources e.g. magazines, social media	9	=1	
Insert into Red Book that is clear and simple, designed by GRTs	10		
Appropriately designed leaflets and verbal personalised information from trusted Health Professional in GP practice	7	=1	=4
Appropriately designed leaflets and verbal personalised information from trusted Health Professional at home	=3	=1	=4
Training for Health Professionals to identify those most concerned about immunisations to discuss their fears and concerns	=4		
***Developing trust and respect***			
Cultural Competence Training for Health Professionals, Frontline Staff and other Service Providers who work with GRTs	=4	=1	3
Named person in GP practice who is trusted by the community for frontline service at reception desk and link to Health Professionals	=3	=1	
***Improving immunisation services***			
Multi-sectorial working led by Health Professionals to raise understanding of cultural issues among professionals in all sectors	5	=1	
Flexible and diverse approach to booking appointments, recall and reminder systems	8	=1	
Flexible delivery of immunisation services to meet specific needs of most socially excluded GRTs, e.g. drop-in clinics, outreach		=1	
Protect funding of specialist roles, e.g. Health Visitor post dedicated to GRT communities	=3	=1	**1**
Improve joined up working and planning between diverse organisations involved in commissioning and delivery of immunisation services			5
Representation from GRT community at meetings of local Immunisation committees	2		
Identify GRTs in health records to record immunisation uptake and tailor support	**1**	=1	2
Improve system of temporary registration at GP practices	6		

*Note.* Independent rankings were based on perceived impact. 1 = greatest impact. Jointly agreed rankings were based on perceived impact, acceptability and feasibility. ^a^Service Providers ran out of time, agreeing 10 key interventions but not ranking them so all recorded as =1

### Stage 2 - Workshops with GRTs and Service Providers

The aim of the workshops was to “co-produce” [29] a prioritised list of interventions to increase immunisation uptake; and to discuss the content and delivery of these interventions for each community. Five half-day workshops were held from November 2014 to September 2015: one workshop in Bristol attended by both the Roma and English Gypsy/Irish Traveller communities, one workshop in York and in London, and two workshops in Glasgow - one each for the Eastern European Roma community and the Scottish Showpeople. Fifty-one GRTs and 25 Service Providers attended a workshop (see Table 3). Prior to commencing each workshop written consent was collected from participants. The workshops with the Roma participants were conducted with the assistance of interpreters (Romanian, Slovak).

**Table 3:** Characteristics of GRTs and Service Providers who attended a workshop

		Bristol Roma	Bristol English Gypsy	Bristol Irish Traveller	York English Gypsy	Glasgow Romanian Roma	Glasgow Slovakian Roma	Glasgow Scottish Showpeople	London Irish Traveller
**GRT All**									
**Total**	**51**	10	2	0	4	7	2	4	11
Mother	18	5	1	0	4	3	1	2	1
Grandmother	13	2	0	0	1	0	0	1	5
Woman no children	4	0	1	0	2	0	0	1	2
Adolescent girl	6	0	0	0	0	0	0	0	3
Father	7	2	0	0	1	4	1	0	0
Grandfather	2	1	1	0	0	0	0	0	0
Male no children	1	0	10	0	0	0	0	0	0
**Service Provider All**									
**Total**	**25**	6			10	4		1	4
Frontline workers	15	3			5	3		1	3
Strategic roles	10	3			5	1		0	1

*Note.* The target sample was 10–12 GRTs from each community across family roles and 3–4 associated Service Providers

In each workshop, following the spoken presentation of the key findings from the interviews by the local researcher, a structured and facilitated two-step process [38] was used to prioritise the interventions. First, GRTs and Service Providers worked in separate groups. The local researcher presented the ideas identified in stage 1. Each idea was written on an A4 sheet of coloured paper and read out to the group with supporting detail from the interviews about the idea. Given that these ideas had emerged from the interview data we were confident that they were acceptable. Participants then discussed and scored each idea in terms of perceived impact (if this idea was adopted how much of a difference would it make to your/the local GRT community in having injections? *1 = no difference to 5 = a lot of difference)*. This ranking did not consider the potential ease or difficulty of implementation and their potential influence on perceived impact). Participants were also asked to identify potential barriers and facilitators to the ideas working in their community. At the end of this session, GRTs and Service Providers had both drawn up a ranked list of intervention ideas based on these impact scores. GRTs and Service Providers then came together and a facilitated discussion, including further discussion on feasibility, was conducted to produce a jointly agreed, prioritised list of five feasible and acceptable interventions which could positively impact on immunisation uptake in their community. As an example, Table 2 presents the independent and joint rankings of the intervention ideas from the York workshop.

### Stage 3 - Cross-community synthesis

Emerging findings from stages 1 and 2 clearly revealed shared rankings, concerns and potential solutions, across communities. For some interventions, high impact rankings were assigned by GRT participants across communities but not by the Service Providers. For others, the reverse occurred. Therefore, stage 3 involved looking across both the independent GRT and Service Provider (impact) rankings and final co-produced, prioritised lists of interventions from each workshop to identify the five most consistently well supported interventions. Interventions needed to be in the top six of the independent rankings by the GRTs and/or Service Providers to be included in the final list. Interventions that were strongly supported but only by particular, usually one or two, communities and their Service Providers were also recorded. These are reported elsewhere [13].

At this stage a detailed description elaborated the nature of each intervention in accordance with the ‘modelling process and outcomes’ step in the MRC guidance on developing and evaluating complex interventions [23]. This included documenting any differences in content and delivery of the intervention required for different GRT communities.

## Results

**Fig. 2:**
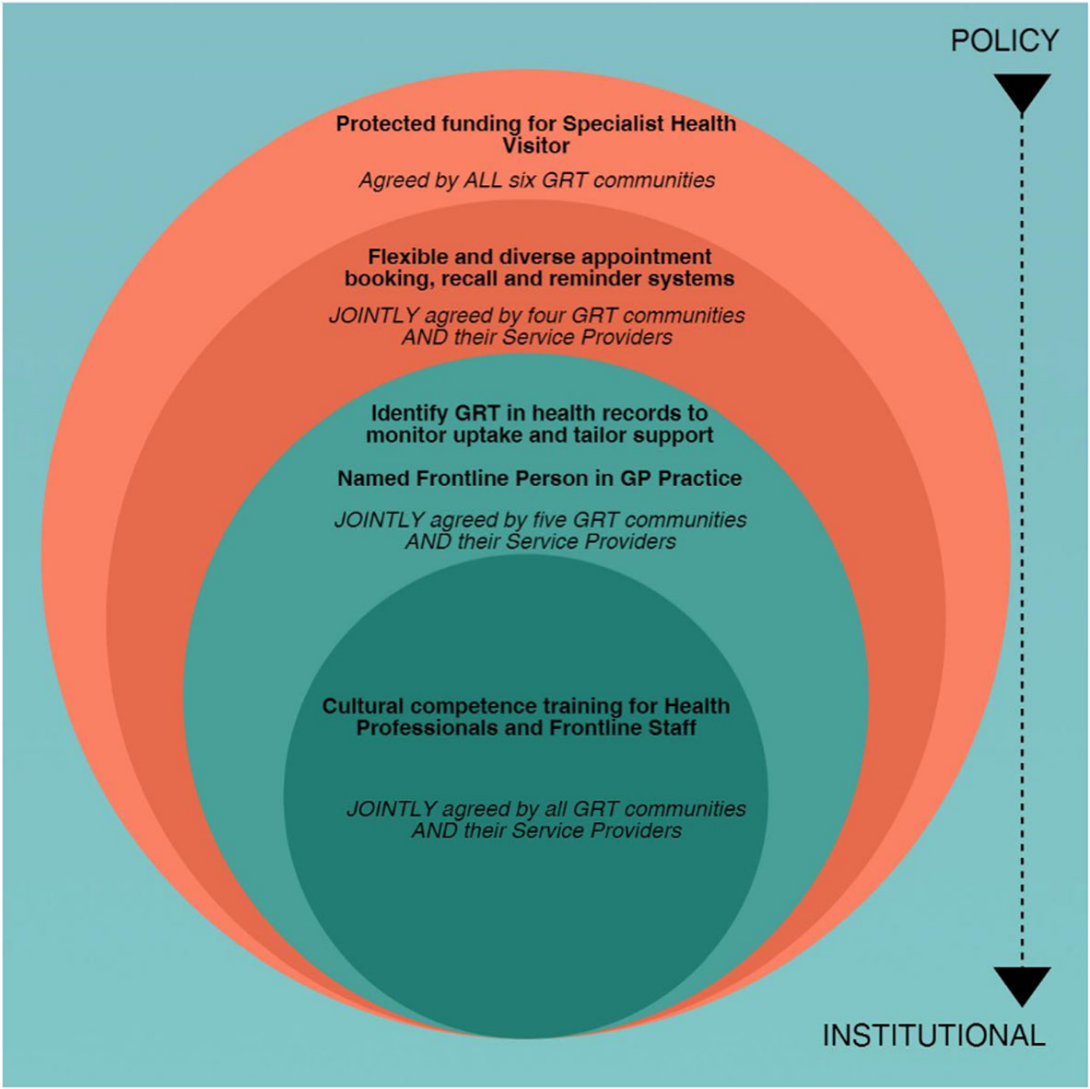
Top priority interventions to improve uptake of immunisation, identified across GRT communities and Service Providers. *Note*: This image was previously published in Jackson, C. et al. (2016). Understanding uptake of Immunisations in Travelling aNd Gypsy communities (UNITING): a qualitative interview study. National Institute for Health Research, Health Technology Assessment, 20(72), 1–176. DOI: 10.3310/hta20720

Five interventions emerged as most consistently supported across GRT communities and/or their Service Providers to improve uptake of immunisation among GRT who are housed or settled on an authorised site (see Fig. 2). The intervention most strongly supported by both GRT communities and Service Providers was ‘Cultural competence training for Health Professionals and Frontline Staff’, illustrated by the smallest circle. The other interventions that were prioritised with less consensus are represented by the circles that increase with size. All five interventions equated to Institutional and Policy levels of influence in the SEM [35].

Detail (components, delivery, differences, barriers) for the five interventions are presented in Table 4. The differences include where an intervention was not considered necessary for a community, for example “named frontline person in GP practice” for the Glasgow Scottish Showpeople; as well how content might differ, for example ensuring that cultural concerns about the HPV and pregnancy vaccines are included in cultural competence training for those working with English Gypsies/Irish Travellers in Bristol.

**Table 4:** Detailed Description of the five prioritised interventions agreed across GRT communities and their Service Providers

Interventions	Components	Delivery	Differences	Barriers
**1: Cultural competence training for Health Professionals and Frontline Staff** (Links with interventions 2 and 3)	National policy intervention to include cultural competence training as a Key Performance Indicator to improve standards and best practice across all GP practices. Cultural competence training of Health Professionals and Frontline Staff on: Cultural norms of different GRT communities towards immunisation in general; these should include positive and negative norms, for example, prioritising children and their good health within the family; Cultural concerns regarding specific immunisations to enable staff to openly and confidently discuss these issues with GRTs; Awareness and understanding of the prejudice that GRTs can face in general; Removal of negative stereotypes towards GRTs and ensure trust and respect is shown towards GRTs; Culturally appropriate methods of collecting ethnicity data from different GRT communities; Cultural understanding of GRT lifestyle and values for interpreters who are often middle class and may not have previously worked with GRTs.	As a minimum standard, training to target GPs, Practice Nurses, Health Visitors, Midwives, Specialist Health Workers and Receptionists. Widespread support warrants national policy and guidance to achieve universal implementation and standards of service.	Culturally informed concerns about pregnancy vaccines and HPV were particularly evident in the Bristol English Gypsy/Irish Traveller community.	Existing resources may be available via some local training schemes but long-term core funding needed to enable consistency of standards and practice.
**2: Identify GRTs in health records to record immunisation uptake and tailor support** (Links with interventions 1 and 3)	Policy mandate (as for 2001 census) for electronic identification of GRTs based on 2011 census which included an English Gypsy/Irish Traveller ethnicity category, with additional categories for Roma and Occupational Travellers; NHS England to provide guidance on codes for GRT identification as part of broader guidance on codes for ethnicity; Central government targets for routine data collection so recording GRT ethnicity becomes part of the ‘data dictionary’; National standards and protocols to provide clarity regarding confidentiality and sharing of information; Improved joined up working and cross-referencing records held by NHS, schools, social services and education, for example the annual school census held by Local Authorities includes a GRT code, local health worker knowledge, postcode data, distinctive surnames; New registration forms for GP practices to include 2011 census classification of ethnicity; Local health professionals to check immunisation status opportunistically to update GRTs’ health records; Local health professionals to encourage effective use of patient held records (Red Book) as an up-to-date immunisation record.	As a minimum, public bodies including GP practices and immunisation datasets to adopt 2011 categories with additional categories for Roma and Occupational Travellers as indicated above. All childhood and adult vaccines should be recorded on the electronic record for each identified GRT. Widespread support and complexity of intervention warrants national policy and guidance to achieve universal implementation and minimum standards for data protection and confidentiality.	Although there is widespread support for this intervention to improve service provision and record keeping, a sensitive approach is needed to take this intervention forward, with particular attention to: Fears of prejudice if identified as GRTs; A general reluctance to self-identify by Romanian and Slovakian Roma; The Romanian and Slovakian Roma community in Glasgow were particularly interested in improving links with health services in Romania and Slovakia so that information on ‘immunisation’ status can be shared across countries. This intervention was not taken to the workshop for consideration by the Glasgow Scottish Showpeople and their Service Providers as it was not supported as a potentially useful intervention based on their interview data. This is consistent with the overarching view of this community as an integral part of the local community with good access to mainstream services.	A lack of national policy and guidance is likely to result in variation in practice between cities. Existing recording systems for data on Romanian and Slovakian Roma immigrants is considered to be poor in Bristol.
**3: Named frontline person in GP practice to provide respectful and supportive service** (Links with interventions 1 and 2)	Existing high levels of trust for individual GPs, Practice Nurses and Health Visitors across all GRT communities provide a strong foundation on which to build this intervention. The named person(s) should be able to provide a consistently positive and culturally appropriate experience for the GRT on his/her arrival at the GP practice. In many cases, this simply refers to being spoken to with respect and politeness whereas in others, GRTs may require support with literacy or language issues to complete a form or identify the appropriate care pathway. Receptionist(s) or Practice Administrators have been identified as potentially suitable to provide this basic standard of service The frontline service should include a timely referral to a Health Professional who has the appropriate training, competency and local knowledge to provide a culturally appropriate immunisation service. Culturally appropriate frontline and health care services within the GP practice will provide continuity of care for any outreach services targeting the most socially excluded GRTs.	Widespread support warrants national policy and guidance to achieve universal implementation and standards of care.	Good practice should be identified and shared with other GP practices. This intervention was not taken to the workshop for consideration by the Glasgow Scottish Showpeople and their Service Providers as it was not supported as a potentially useful intervention based on their interview data. This is consistent with the overarching view of this community as an integral part of the local community with good access to mainstream services.	Creation of a culturally appropriate and accessible primary care service will be of potential benefit to all childhood and adult health services for GRTs.
**4: Flexible and diverse systems for booking appointments, recall and reminders** (Links with interventions 2 and 5)	The core component of this intervention is inclusion of a SMS text-based approach for immunisation recall systems, booking the appointment and regular reminders, in addition to existing letter systems. A combined system of both letter and SMS text communication is required due to differing literacy needs and fluctuating credit levels on phones. The core SMS text intervention should be demanded as standard through national policy and potentially as a quality indicator of ‘access and flexibility’ as per disability indicators. Appointments within 1–2 days of booking are more likely to be attended than appointments booked for a fortnight’s time due to some GRTs’ broad concepts of time and difficulty with the commitment of a fixed appointment. Existing annual reminder systems, e.g. for the flu vaccine, are considered to work well and should remain an integral part of any adapted system.	Widespread support warrants consideration for national policy and guidance to achieve universal implementation and standards of care.	This intervention was not discussed by SPs working with the Roma community in Glasgow. A more flexible system for conducting the immunisations e.g. out-of-hours appointments, was not supported. This intervention was not ranked within the top six priority interventions by GRT communities and SPs in York and London (ranked in top 8). SPs in York ranked this as a top priority. GRTs working in low paid employment, for example, Romanian and Slovakian Roma in Bristol, are often working long and antisocial hours, making it difficult to attend for immunisations within usual clinic times.	Health Visitors or other Health Professionals working with GRT communities could usefully provide additional, face-to-face reminders where possible. Good practice should be identified and shared to other GP practices.
**5: Protected funding for Health Visitor specialising in GRT health including immunisation** (Links with interventions 1 and 2)	The specialist service provides an important outreach component to target those families who do not access mainstream services. The outreach service increases access to culturally appropriate and personalised information and service support, referral to immunisation services within the GP practice and improved linkages between GRT families and the GP practice. Practice based services delivered by a specialist Health Visitor identified as important to improve uptake of immunisation include drop-in clinics for specific population groups within GRT communities, e.g. adolescent girls and/or specific vaccines, e.g. HPV; out-of-hours appointments. A specialist Health Visitor has detailed local knowledge of existing and new families within the GRT community. This has typically resulted in a targeted and timely service and would inform identification of GRTs in their health records for improved monitoring of uptake (see Intervention 2).	Widespread support across all GRT communities warrants national policy and guidance to achieve universal implementation and minimum standards of care.	Continuity of high-quality care from a trusted service provider, typically a Health Visitor, has been identified as one of the most important and valued services by all GRT communities.	Loss of protected funding for this post in the past. Additional resources are required with appropriate policy guidance to prioritise this service within the face of local budget constraints.

## Discussion

In this paper, we describe the approach taken to identify potential interventions that could increase uptake of immunisation in GRT communities which are housed or settled on an authorised site. We present the five interventions that emerged as most consistently supported across GRT communities and/or their Service Providers. We reflect here on the identification process and consider its strengths, challenges, and limitations. We then discuss the interventions in the context of existing practice, policy and research.

The key strength of our approach was that we used established methods for robust intervention development [23, 25, 29, 38]; we identified desired intervention outcomes (using intervention mapping), drew on evidence (interviews with the six GRT communities and their Service Providers), employed theory (SEM [35]), tailored the interventions to different segments of the population (different GRT communities) and worked in partnership with GRTs and their Service Providers to ensure that the final prioritised interventions were informed by their views and experiences. This grassroots experience also generated detailed insight into the barriers and facilitators to potential implementation of the five prioritised interventions which can inform their future development and evaluation [23]. Application of the SEM to “real-life” recommendations was useful in determining the level at which prospective interventions or changes in practice might best be targeted. The workshops were informal and highly interactive events with many GRTs and Service Providers commenting that this was the first time they had been given the opportunity to talk to each other about services. GRT participants were generally adept in highlighting applied and practical implications for practice. For example, in discussing the intervention “flexible and diverse appointment booking, recall and reminder systems” the Glasgow Scottish Showpeople offered the view that shared mailboxes in static caravan yards were a barrier to the timely receipt of immunisation appointments, and suggested that text messaging might be a preferable method. In some workshops, there was a lack of concordance in the independent impact rankings by GRTs and Service Providers (see “Good information from non-NHS sources” in Table 2) and therefore coming together and hearing a different perspective to generate the prioritised list of interventions with shared ownership was essential. A challenge of the workshops was completing the two steps of prioritisation in the time available. This meant that sometimes the first step discussions (when GRTs and Service Providers worked independently) were cut short and the interventions perceived to have greatest impact were agreed but not ranked (as occurred for the Service Providers in York, see Table 2). This was resolved in the second, joint-working, step, but it did mean that there were some missing data for the cross-synthesis. When this occurred, data from the remaining rankings were used.

It is important to consider how transferable the finalised list of interventions is to (1) other GRTs/Service Providers within these six communities and (2) other GRT communities in the UK. With respect to (1) we are confident the initial selection of intervention ideas (in stage 1) reflected wider community views as these were based on interviews with 174 GRTs across family roles and immunisation experiences; and 39 Service Providers in both frontline and strategic roles [12–14]. As such we are confident in the acceptability of the final interventions. We did not meet our target sample for the workshops for all six communities however, the transparent approach regarding the level of agreement within and across GRT communities and Service Providers for the five interventions (Fig. 2) is reassuring. In addressing (2) based on the above mentioned interviews [12–14], evidence suggests that these GRT communities share immunisation views, norms and experiences with communities of the same descent (e.g. other Irish Traveller communities) [12–17, 39–43]. We therefore believe that the five interventions are relevant to members of other GRT communities of English, Irish, Romanian/Slovakian Roma and Scottish Showpeople descent who are housed or settled on an authorised site. We do not know if these interventions would be prioritised by GRT communities who live on the roadside or in unauthorised encampments.

The final five prioritised interventions were all “upstream” interventions [44] and focused on addressing barriers of access to immunisation services rather than concerns about the safety or beliefs about the necessity of vaccines. Workshop participants were clear that these interventions were complementary and likely to have greater impact if delivered together. This is consistent with findings of a recent review [19] that locally developed, multicomponent interventions are most effective in reducing inequalities in immunisation uptake. Local implementation of the five interventions will need to reflect some differences in content and delivery between communities (see Table 4).

Interestingly, these proposed interventions were not necessarily new, nor were they unique to the needs of GRTs. The UK 2010 Equality Act [45] and other guidance for working with GRTs [1, 4, 16, 17, 32, 46] and other minority groups [47] acknowledge the importance of cultural competence training to develop Service Provider understanding of different cultures and the impact of discrimination with a view to building trustful relationships to enhance care. Strong monitoring and surveillance systems are an accepted component of a good immunisation programme [19, 48]. Specific to GRTs, is the need to include an ethnicity category that enables them to self-identify. In England, the 2011 national census category “Gypsy or Irish Traveller” is not routinely used in NHS health systems, for example in GP practices and Child Health Information Systems [2, 49, 50]. Furthermore, this category does not enable Roma or Occupational Travellers to self-identify [2, 4], and when GRTs are not identified as distinct ethnic groups, understanding of their barriers to healthcare provision is limited [2, 4, 51]. Capturing this information would mean that patterns of health services utilisation, including immunisation, could be monitored to identify health inequalities [49] and tailor service provision to reduce inequalities [2, 4, 49, 52]. The fear of discrimination may deter GRTs from self-identifying [4] again reinforcing the importance of delivering these interventions as a package. The combined effect is to create an environment in which GRTs feel safe to disclose their ethnicity.

The UK National Institute for Health and Care Excellence [53] and the World Health Organisation [48] recommend offering flexibility of booking and attending immunisation appointments to ensure that healthcare is accessible [3, 4, 30, 54]. Effective recall, reminder and appointment systems for childhood and adult vaccinations [21, 55] can be tailored to meet the specific needs of each GRT community. For example, rather than delivering specialist immunisation services to a community, it may be better to improve signposting and access to mainstream provision [33, 43, 54]. Finally, an important finding - which is rarely formally evaluated [33] – confirms that GRTs value the role that Specialist Health Visitors play in facilitating access to health services including immunisation [1, 33, 56]. Unfortunately, insight from our interviews with Service Providers [12, 14] suggested that these roles are no longer funded in all the study locations, meaning that access is inequitable.

## Conclusions

The five prioritised interventions were Institutional and Policy level interventions, reinforcing the key role of GP practices, frontline workers and wider NHS systems on improving immunisation uptake. All five interventions have potentially broader applicability than GRT communities alone. The complementary nature of these interventions suggests that their impact would be enhanced if delivered as a combined package. Local implementation must reflect community differences. We have used robust intervention development and co-production methods which could usefully be applied to other communities where uptake of immunisation is a concern.

## Data Availability

The datasets generated during and/or analysed during the current study are not available due to concerns about deductive disclosure.

## Abbreviations

GP: General Practitioner
GRT: Gypsy, Roma and Traveller
MRC: Medical Research Council
NHS: National Health Service
SEM: Social Ecological Model
UK: United Kingdom
UNITING: UNderstanding uptake of Immunisations in TravellIng aNd Gypsy communities
